# Relationships between Spatial Metrics and Plant Diversity in Constructed Freshwater Wetlands

**DOI:** 10.1371/journal.pone.0135917

**Published:** 2015-08-21

**Authors:** Erika C. Brandt, John E. Petersen, Jake J. Grossman, George A. Allen, David H. Benzing

**Affiliations:** 1 Environmental Studies Program, Biology Department, Oberlin College, Adam Joseph Lewis Center, 122 Elm Street, Oberlin, Ohio, United States of America; 2 Department of Ecology, Evolution and Behavior, University of Minnesota, 100 Ecology Building, 1987 Upper Buford Circle, Saint Paul, Minnesota, United States of America; Shandong University, CHINA

## Abstract

The diversity of plant species and their distribution in space are both thought to have important effects on the function of wetland ecosystems. However, knowledge of the relationships between plant species and spatial diversity remains incomplete. In this study, we investigated relationships between spatial pattern and plant species diversity over a five year period following the initial restoration of experimental wetland ecosystems. In 2003, six identical and hydrologically-isolated 0.18 ha wetland “cells” were constructed in former farmland in northeast Ohio. The systems were subjected to planting treatments that resulted in different levels of vascular plant species diversity among cells. Plant species diversity was assessed through annual inventories. Plant spatial pattern was assessed by digitizing low-altitude aerial photographs taken at the same time as the inventories. Diversity metrics derived from the inventories were significantly related to certain spatial metrics derived from the photographs, including cover type diversity and contagion. We found that wetlands with high cover type diversity harbor higher plant species diversity than wetlands with fewer types of patches. We also found significant relationships between plant species diversity and spatial patterning of patch types, but the direction of the effect differed depending on the diversity metric used. Links between diversity and spatial pattern observed in this study suggest that high-resolution aerial imagery may provide wetland scientists with a useful tool for assessing plant diversity.

## Introduction

Plant species diversity is valued, in part, for its potential effects on ecosystem functions, such as primary productivity and nutrient cycling (e.g., [1–3]). Spatial diversity, or the spatial patterning of environmental characteristics, such as plant cover type, is also thought to have important implications for function (e.g., [4, 5]). For example, it is well known that at the landscape scale, riparian wetlands have a greater influence on stream water quality than non-riparian wetlands (e.g., [6]). At a smaller spatial scale, within wetlands, functional attributes such as nitrate removal [7] and nutrient release rates [8] have also been found to correlate with spatial patterns in plant cover. Despite interest in these two types of diversity and their respective links to ecological function, relatively few studies have examined the relationship between plant species diversity and spatial patterning of plant cover, particularly at smaller scales (e.g., [9]).

Although ecologists understand that pattern and process are related at all scales (e.g., [10]), most spatial analyses to date have been conducted at the scale of multi-hectare landscapes, where spatial data are available from remote sensing technology. Such analyses tend to focus on characterizing and assessing the general importance of spatial patterns in vegetation and other land cover types rather than the spatial arrangement of specific species. However, as advances in remote sensing technology, such as drones, continue to improve our ability to detect fine-scale pattern, the distinction between analysis of general land cover type and assessment of species effects blurs. Already in forest ecosystems, imagery obtained from satellites and aircraft is of sufficient resolution that individual trees can often be distinguished and identified to the species level. As remote sensing technology continues to improve, spatial analysis will enhance species identification in ecosystems such as wetlands, which may possess fine-scale, sub-meter variability in plant cover.

Improving our understanding of the relationships between plant species diversity and spatial pattern may be particularly important and useful in wetlands, where individual species reflect important differences in microhabitat and may exhibit distinct functional attributes. Achieving high native species diversity is also often an explicit goal of wetland restoration [11, 12]. Improving our understanding of the relationship between plant diversity and remotely sensed spatial patterning of plant cover could therefore also enhance our ability to track the success of restoration projects.

Our goal in this study was to quantify relationships between species diversity and spatial heterogeneity of wetland plants under controlled conditions. Over a five year period following planting, we assessed spatial patterning of vegetation and plant species diversity in six constructed wetland ecosystems that had been managed to achieve different levels of diversity. We expected that measures of plant species diversity in individual wetlands would be related to measures of spatial diversity, such as cover type diversity and aggregation.

## Materials and Methods

### Study site

Research was conducted in an experimental restored wetland system located on post-agricultural land in the Lake Erie drainage basin of northeast Ohio on property owned by Oberlin College (41°17'38” N, 82°13'03” W, the authors can be contacted regarding future permission to access the site). Poor drainage and surface flooding are characteristic of this region owing to level topography and post-glacial clay loam soil. From 1970 to 2000, mean precipitation was 92 cm/year with monthly temperature averages ranging from -5°C in January to 22°C in July [13]. Prior to settlement, post-glacial beech-maple (*Fagus grandifolia* Ehrh.-*Acer* sp. Marshall) forests and swamp forests dominated this region [14]. Before wetland restoration, this site was in a commercial corn-soy rotation for multiple decades and then lay fallow for two years.

**Fig 1:**
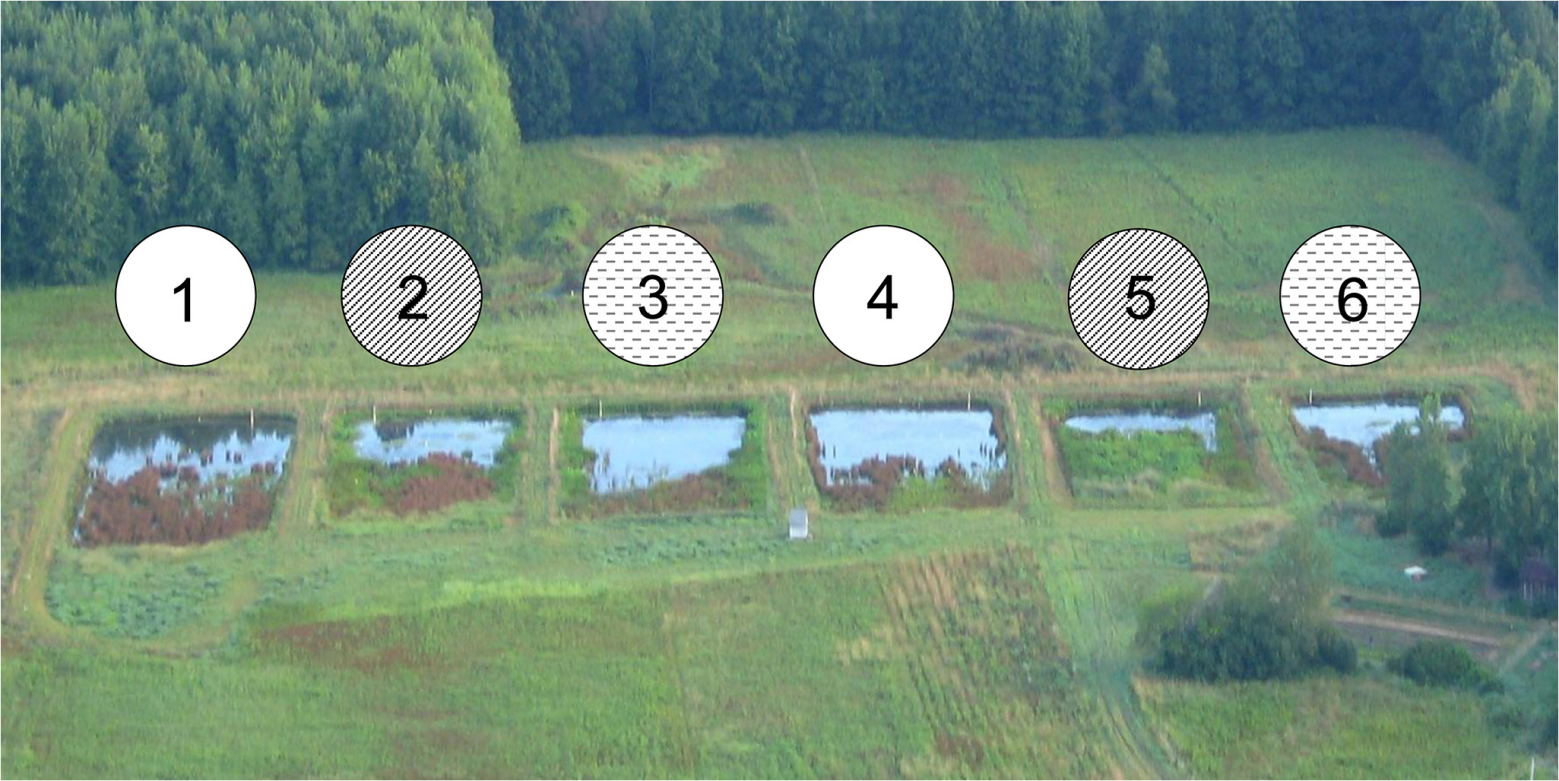
Aerial view of experimental wetland cells taken in 2006. “High-intensity” cells 2 and 5 were subjected to repeated plantings starting in 2003. “Low-intensity” cells 3 and 6 received initial planting and replanting the subsequent year. Cells 1 and 4 were unplanted. Cells 2, 3, and 4 were fertilized in 2010 and 2011.

**Fig 2:**
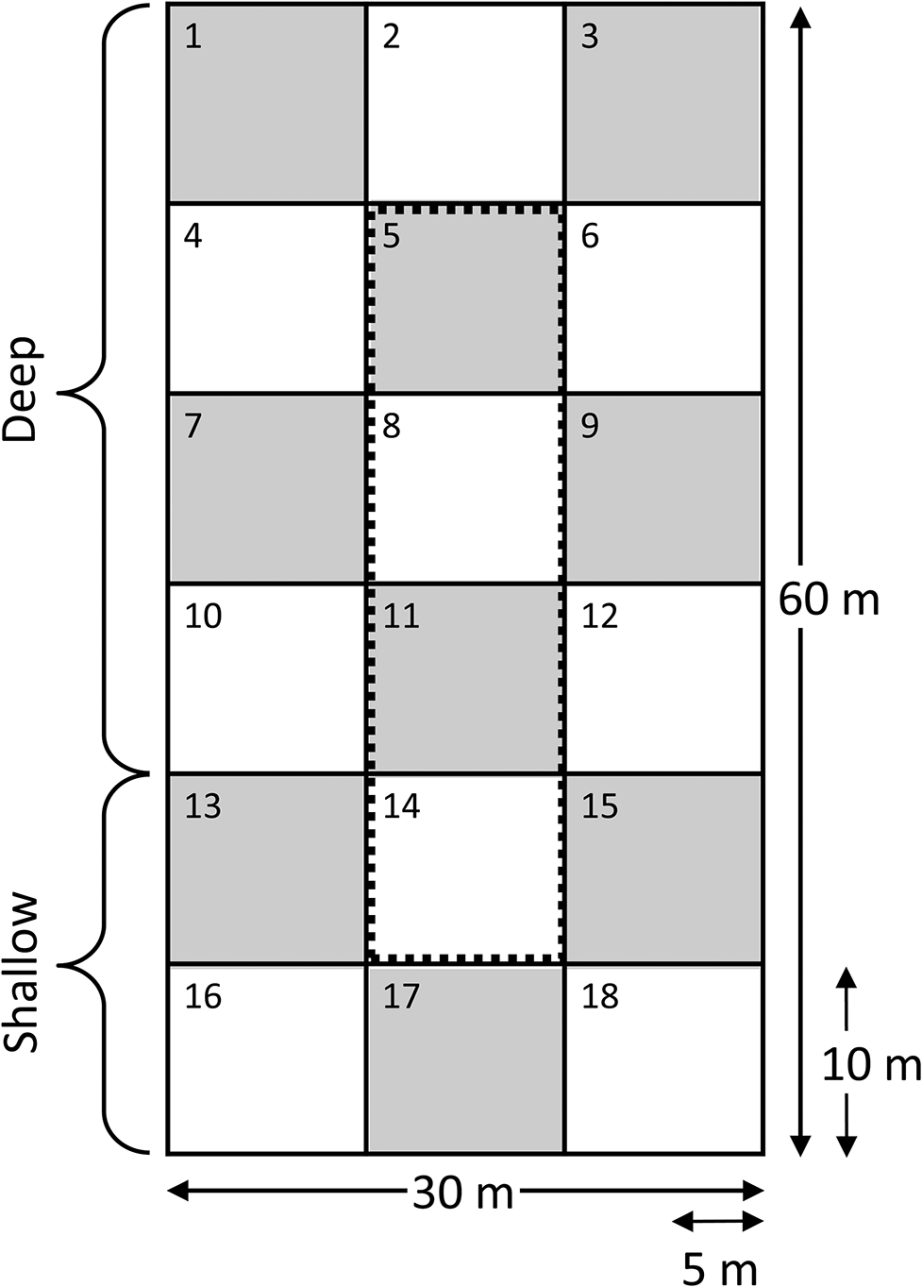
Sampling quadrats and zones for analysis in each wetland cell. Permanent rebar posts marked the edges of 10 x 10 m quadrats in each cell. Quadrats in which the annual species surveys were conducted are delineated in grey and identified with odd numbers. Analyses were conducted at the level of whole cells and by zone. The “central” zone (quadrats inside the dotted line) has no adjacency to upland regions (quadrats 5, 8, 11, and 14). The “edge” zone contains or is bordered by upland terrain outside of the cell (all remaining quadrats). The “deep” zone encompasses regions containing deeper water (quadrats 1–12), while the “shallow” zone is comprised of regions that were seasonally dry (quadrats 13–18).

Wetlands were constructed in July 2003 by blocking a tile drainage system, excavating depressions, and building berms on the downslope northern side in a series of north-south divisions to create six adjacent, uniform, hydrologically-isolated, rectangular wetland “cells” (Fig 1). Each cell is 60 x 30 m. Maximum depth is approximately 1.5 m at the north end (designed to retain water in dry years) and slopes to seasonally wet meadow on the south end. Adjustable weirs in each basin control maximum water level. The watershed draining into each cell is between 0.5 and 1 ha, dominated by annual grasses that were mowed every spring during the first four years of restoration. A permanent grid marked by rebar posts was established within each cell to create a fixed reference system for sampling and spatial analysis (Fig 2). For the work reported in this paper, the finest resolution of on-the-ground species identification took place in eighteen 10 m x 10 m quadrats marked by rebar at each corner in each cell (Fig 2).

### Experimental system, treatments and management

The analyses described in this manuscript were conducted on data obtained from an experimental system that was designed for a separate study assessing the effects of planting treatments on species diversity, and then the effects of the resulting species diversity on ecological function [15]. The study described in the present manuscript, however, is restricted to addressing how spatial measures of diversity assessed through aerial photography relate to direct inventories of diversity on the ground. The particular treatments and definition of experimental units in the original experiment (whole wetland cells) are not the same as the relevant experimental units for analysis in this study (quadrats). An understanding of the initial experimental design (explained in the next two paragraphs) is important, however, as it provides context for understanding how and why different plant species diversity levels were generated in different quadrats.

Wetland cells were initially subjected to a planting treatment in years one to four, and then to a nutrient addition treatment in years seven and eight. Three duplicated planting treatments were used (Fig 1). Cells 1 and 4 were left unplanted; colonization occurred through natural recruitment alone. A “high-intensity” treatment (cells 2 and 5) and “low-intensity” treatment (cells 3 and 6) were extensively planted in 2003 with a mixture of locally-collected and commercially-acquired seeds (11 species total) and vegetative propagules of native wetland species (10 species, Table 1). Vegetative propagules that did not survive initial planting were replanted in both high- and low-intensity treatments in summer 2004. In the high-intensity treatment, missing propagules were replanted again in summers 2005 and 2006. Seeds were not re-sown in either treatment, as they were less successful at establishing than vegetative propagules.

**Table 1:** Propagules planted in low- and high-intensity planted cells in 2003.

Propagules	Amount Planted	Source
**Vegetative**		
*Saururus cernuus*	8 individuals/cell	Local
*Peltandra virginica*	17 individuals/cell	Local
*Acorus americanus*	16 individuals/cell	Nursery
*Carex stricta*	16 individuals/cell	Nursery
*Spartina pectinata*	16 individuals/cell	Nursery
*Sagittaria latifolia*	17 individuals/cell	Local
*Sagittaria latifolia*	16 individuals/cell	Nursery
*Pontedaria cordata*	2 individuals/cell	Local
*Pontedaria cordata*	4 individuals/cell	Nursery
*Iris versicolor*	16 individuals/cell	Nursery
*Sparganium americanum*	15 individuals/cell	Local
*Nymphaea odorata*	10 individuals/cell	Local
**Seed**		
*Carex frankii*	215 g/cell	Nursery
*Carex vulpinoidea*	215 g/cell	Nursery
*Scirpus validus*	55 g/cell	Nursery
*Juncus torreyi*	25 g/cell	Nursery
*Juncus effusus*	26 g/cell	Nursery
*Hibiscus mocsheutos*	8.5 g/cell	Local
*Asclepias incarnata*	12.5 g/cell	Local
*Decodon verticillatus*	2.7 g/cell	Local
*Rosa palustris*	1.25 g/cell	Local
*Lobelia cardinalis*	0.9 g/cell	Local
*Cephalanthus accidentales*	0.42 g/cell	Local

Species planted originally as vegetative propagules were replanted in high-intensity cells in 2004, 2005, and 2006.

The second treatment was a nutrient addition designed to simulate runoff from a watershed dominated by conventionally-managed corn and soy agriculture. In the summers of 2010 and 2011, commercial nitrogen (N) and phosphorus (P) fertilizer (urea and mono ammonium phosphate) was spread in one cell from each planting treatment. Application rates simulated a wetland:watershed ratio of 0.04. Details related to application of fertilizer and the functional response are discussed in a separate paper [15]. Nutrient addition had no detectable effect on plant species diversity and we saw no evidence that it affected spatial patterning of vegetation. We therefore do not treat post-fertilization data differently from pre-fertilization data in the present study.

Two invasive plants, reed canary grass (*Phalaris arundinacea* L.) and cattail (*Typha angustifolia* L. and *T*. *latifolia* L.), and one animal, muskrat (*Ondatra zibethicus*), were controlled during the study period. Reed canary grass was controlled as part of the experiment with the herbicide glyphosate in dry regions of wetlands (2004–2006; minimal overspray entered the water) and selective removal and mowing (2004–2007). Cattail was manually removed during the summers of 2004 and 2005. Cattail removal stopped once populations remained relatively low (<1% total coverage). Reed canary grass, however, continued to dominate some regions of cells once removal ceased. In total, 36.5 person-hours were expended weeding in 2004, 16.3 hours in 2005, 13.8 hours in 2006, and then weeding ceased in subsequent years. Muskrats invaded the cells in 2004 and inflicted damage through herbivory and extensive excavation of berms.

Control of muskrats was initiated by site managers, independently of the experiment, and took place on other wetlands on the 28 ha parcel of land on which the experiment wetlands were located. Conibear traps were deployed, primarily during the winter by a professional trapper who was granted access to this and other sites. However, muskrat population and resulting herbivory persisted throughout the remainder of the experiment. According to records shared by the trapper, the greatest number of muskrats caught in one season was 25 (in 2012). Muskrats were active in all cells and freely moved among the cells and in the surrounding landscape. Due to the inherent challenges of monitoring a dynamic population, we were not able to methodically quantify the distribution of muskrats herbivory and excavation throughout cells. That said, according to the trappers records, more muskrats were typically caught in cells 3 and 4, and fewer in cells 1 and 6, suggesting that abundance was unrelated to experimental treatment (Fig 1). Since the decision to trap was made by site managers with protocol determined by site managers and not by researchers and since muskrats were not an explicit component of this study and were controlled by a commercial and permitted trapper for his own benefit using standard and legal practices, no IACUC permission was sought.

The developmental trajectory of the wetlands, the effects of planting on species diversity and the effects of diversity on ecological function are all considered in detail in a separate manuscript [15]. The present study considers only the relationship between spatial and direct measures of species diversity and disregards any questions related to how diversity was attained or whether it has an impact on function.

### Data collection and processing

Plant species diversity was surveyed annually from 2004 to 2012 in late July or early August. However, only data from 2008 to 2012 were used in our analyses, as this time frame corresponds to our spatial pattern data. Our methodology was based on Ohio EPA procedures [16, 17]. Within each cell, we surveyed nine 10 x 10 m quadrats covering half of the total area of each cell in a checkerboard pattern (Fig 2, species assessments were conducted in numbered quadrats which are also marked in grey). We recorded percent cover of all plant species within each quadrat. Plant identification and nomenclature followed Gleason and Cronquist [18]. Percent cover was determined according to the system of Peet, Wentworth and White [16].

In each of the six wetland cells, a common designation was used to assign quadrats to four, sometimes overlapping, “zones” based on location (Fig 2). The “central” zone is comprised of all quadrats that have no adjacency to upland regions while the “edge” zone contains quadrats that are bordered by upland terrain outside of the cell. The “deep” zone encompasses all quadrats containing deeper water, while the “shallow” zone is comprised of all quadrats that were seasonally dry.

We assigned attributes to each plant species present in the wetlands using the classification scheme published by Ohio EPA [19]. Species were classified as either native or non-native to Ohio, and as obligate wetland, facultative wetland, facultative (equally amenable to either upland or wetland habitat), facultative upland, or obligate upland. In our analyses of plant species diversity, we treated all obligate wetland, facultative wetland, and facultative species as “wetland species”.

In preliminary analyses, we calculated a range of different diversity metrics at several scales (i.e., treatments, whole cells, zones within cells, and individual quadrats) for all species prevalence data, as well as subsets of the original dataset that included only certain species (native species, wetland species, species that are both native and wetland, and exotic species). General patterns were similar for many of the diversity metrics calculated. For reasons of parsimony, we focus on two measures of plant species diversity in this paper (Table 2). The Floristic Quality Assessment Index (FQAI) and Shannon-Wiener diversity of native species (native SWD) both measure alpha-diversity [20], meaning diversity without reference to the spatial arrangement of plants within the unit evaluated.

**Table 2:** List of abbreviations used to refer to species diversity and spatial metrics.

Abbreviation	Full Name
**Species Diversity Metrics**	
SWD	Shannon-Wiener Diversity
FQAI	The Floristic Quality Assessment Index
**Spatial Metrics**	
SHDI	Shannon’s habitat diversity index
SHAPE_AM	Area-weighted mean patch shape index
CONTAG	Contagion

Over the nine years during which species inventories were conducted we observed 98 total plant species in the wetlands. Ninety-six of these were identified at least once in the planted cells, and 77 in the unplanted cells. To calculate native SWD, we excluded non-native species from the original dataset based on Ohio EPA classification of species attributes [19]. Fifty-five native species were included, 54 in planted and 42 in unplanted. We used a multivariate statistical package (Kovach Computing Services; Pentraeth, Wales) to calculate native SWD [21, 22]. This package calculates SWD using the standard formula:

$$\text{SWD}\;=\;-\sum_{i=1}^{s}p_{i}*\;\text{ln}\left(p_{i}\right)$$ (1)

where S is the number of species and *p*_*i*_ is the percent cover (calculated by averaging range of percentages within a given cover class) in the surveyed area attributable to species *i*. We calculated native SWD for each quadrat assessed.

FQAI is a specialized species richness metric. Like basic species richness, it does not consider abundance data, and can thus be skewed by non-uniform abundance of species in a community [23], which may limit its usefulness as a management tool [24]. However, FQAI contrasts with basic species richness in that it incorporates a “coefficient of conservativism” (*C)* value for each species. *C* is a classification attribute used to weight the relative value of species in plant species diversity assessments. We used the weighting values of Ohio EPA [19], which assign highest *C* values to endemic species of Ohio that have narrow ranges and low or zero values to exotic or generalist species. While native upland species are assigned higher *C* values than non-native upland species, species endemic to wetland habitats are given the highest *C* values, making this a useful classification attribute for assessing wetland plant diversity [25, 26]. FQAI is a widely used diversity metric in studies of wetland plant communities [27–29], as its emphasis on “quality” of plant species makes it a useful indicator of the desirability of plant species composition in wetland ecosystems [30]. We calculated FQAI scores for quadrats following the methods of Ohio EPA [19], which define FQAI as:

$$\text{FQAI}=-\sum_{i=1}^{S}\frac{C_{i}}{S^{0.5}}$$ (2)

where *C*_*i*_ is the coefficient of conservativism for species *i* and *S* is the total species richness of the area being evaluated.

**Fig 3:**
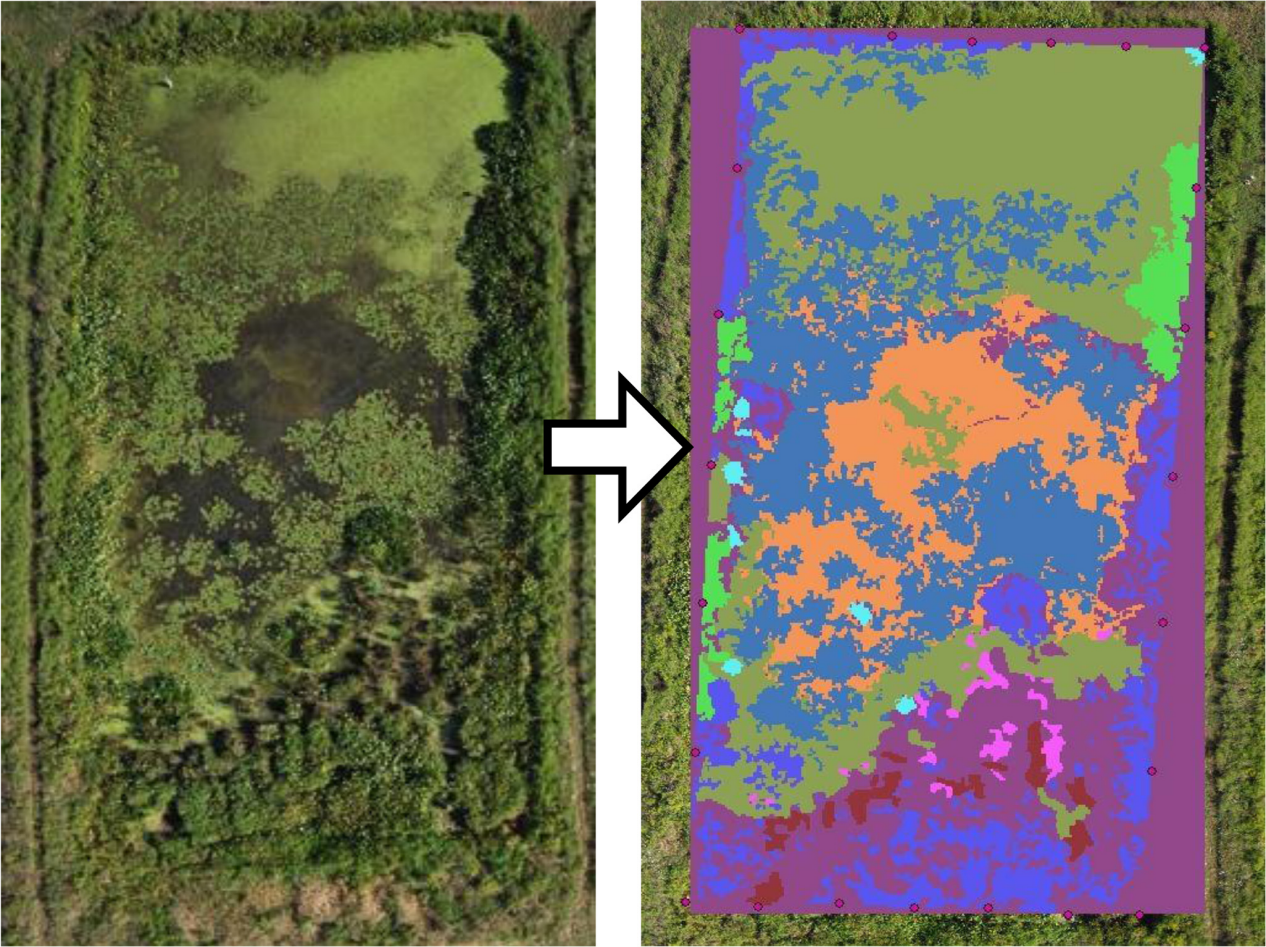
Aerial photograph and subsequent delineation of cover types. This example is of cell 3, a high-intensity planted cell, from a photograph taken in 2010.

Low altitude aerial photographs of vegetation in the cells were taken from 2008 to 2012 in late August using a remote controlled camera mounted on a large helium-filled balloon (e.g., [31]). The balloon was controlled manually from the ground with kite strings and the camera rig was controlled by radio. Aerial photographs were taken of each cell in its entirety in the visible spectrum only and have a sub-meter resolution. Close-up photographs of the cells were taken as well, and used along with diversity surveys for ground-truthing when necessary. The low altitude photographs were imported into ArcMap (ESRI 2011) and georeferenced to GPS points taken at each node of the rebar grid (Fig 3). Polygons representing areas of 11 different cover types were delineated. These included patches of the plant species *Lemna/Wolfia* spp., *Juncus effusus*, *Nymphaea odorata*, *Malva moschata*, *Peltandra virginica*, *Phalaris arundinacea*, *Pontedaria cordata*, *Polygonum* spp., and *Sagittaria latifolia*. Open water and open land were also delineated for each photograph. Some areas occupied by these different cover types (polygons) were delineated manually, while others were first assigned automatically using the Maximum Likelihood Classification tool in ArcMap 10 and then corrected by hand.

In order to minimize human error from hand delineation, each patch was delineated in fine detail, then aggregated and simplified by standard normalization parameters at a more relaxed degree of precision. Polygons were first aggregated (aggregation distance = 0.1 m, minimum area = 0.1 m², minimum hole = 0.25 m²), then simplified (simplification algorithm = point_remove, maximum allowable offset = 0.05 m, minimum area = 0 m, keep collapsed points = no). Polygons were converted to raster (grid) files, then to text files and imported into FRAGSTATS [32].

Spatial metrics of the wetland landscapes were computed in FRAGSTATS. Spatial pattern was assessed for each of the nine quadrats within each cell. In a preliminary analysis, we computed multiple different landscape spatial metrics, several of which were highly correlated with each other. We chose three that that we felt characterized distinct aspects of spatial pattern, including shape, distribution, and diversity of patches of different cover types, each of which is potentially important to ecological function. The three spatial metrics used to explore relationships between plant spatial pattern and plant species diversity were Shannon's habitat diversity index (SHDI), area-weighted mean patch shape index (SHAPE_AM), and contagion (CONTAG; Table 2). Each measure is explained in the paragraphs below.

SHDI is a combined measure of cover type richness and evenness in a landscape. Higher values indicate higher diversity and more even distribution of cover types, while lower values indicate lower diversity and less even distribution. SHDI considers the proportion of a landscape occupied by patches of different cover types, but not the spatial configuration of those patches within the landscape. It is thus very similar to the standard ground-based Shannon-Wiener index used to quantify species diversity, but quantifies diversity based on the smaller number of cover types identified through aerial photography and on total area occupied rather than percent cover within an area. In this study, SHDI was calculated based on the 11 cover types distinguished from aerial photographs, versus the 98 distinct species identified in our species diversity inventories.

SHAPE_AM is a metric that measures the mean complexity of patch shape within a landscape. To accomplish this, the actual shape of each patch is compared to a standard (square) shape of the same area, and SHAPE_AM quantifies the degree to which its edge-to-area ratio differs. An advantage of using SHAPE_AM over other similar metrics like perimeter-to-area ratio is that it is standardized to be independent of patch size.

CONTAG is a metric that measures how clumped patches are within a landscape. It quantifies both patch interspersion (the intermixing of patches of different cover types) and dispersion (the spatial distribution of a given cover type). Lower CONTAG values indicate landscapes made up of many small, dispersed patches, while higher CONTAG values indicate landscapes with relatively fewer, larger, and more contiguous patches. Descriptions of spatial metrics provided above are adapted from FRAGSTATS documentation. More detailed descriptions of metrics, discussion of their usefulness, and their formulas can be found in FRAGSTATS documentation [32].

To analyze the relationship between plant species diversity and spatial pattern, we constructed a mixed linear model in R (R Core Team, 2012) using the package *lme4* [33]. Our data contains multiple observations of the same quadrats over several years (i.e., one observation of quadrat X in 2008, one in 2009, etc.); to account for this non-independence issue, we considered year of observation as a factor acting on spatial pattern (Eq 4). Another non-independence issue with our data is that the 36 quadrats under observation are not independent samples, since they are nested within cells (i.e., nine quadrats in cell 1, nine in cell 2, etc.); to account for this, we also made nested location, or the positioning of quadrats within zones within cells, a factor acting on spatial pattern (Eq 4). First, we constructed a null model that assumes that plant species diversity does not affect spatial pattern; in this model, species diversity, year, and location are all described as random effects. Written in standard matrix notation, the equation for the null model is:

$$y\;=\;X\beta +Z_{1}\mu_{1}+Z_{2}\mu_{2}+Z_{3}\mu_{3}+E$$ (3)

Here, *y* is the dependent variable (spatial pattern) and *X* is the variable conveying fixed effects, *β*, onto *y*; in this null model, we assume that all effects are random, so *X* equals zero. *Z*_1_ (species diversity), *Z*_2_ (year), and *Z*_3_ (nested location) are variables conveying random effects *μ*_1_ and *μ*_2_ onto *y* and *E* is the error term that captures all random error, or discrepancies between the outputs predicted by the model and our actual data that are not explained by other factors. In R, the null model is written:

spatial pattern∼(1|species diversity)+(1|year)+(1|cell/zone/quadrat) (4)

In this representation of the null model, spatial pattern equates to *y* (Eq 3), the term (1 | species diversity) equals *Z*_1_, (1 | *year*) equals *Z*_2_, and (1 | *cell/zone/quadrat*) equals *Z*_2_. The expression *cell/zone/quadrat* captures the nested structure of quadrat locations, in which quadrats are located within zones, which are located within cells.

In contrast to this null model, our working model describes species diversity as a fixed effect acting on spatial pattern. In the working model, year and location act as random effects with the potential to interact with species diversity to further affect spatial pattern. Written in standard matrix notation, the equation for the working model is:

$$y=X\beta +Z_{1}\mu_{1}+Z_{2}\mu_{2}+E$$ (5)

Here, *y* is the dependent variable (spatial pattern) and *X* is the independent variable (species diversity) conveying fixed effects, *β*, onto *y*. *Z*_1_ (year) and *Z*_2_ (nested location) are variables conveying random effects *μ*_1_ and *μ*_2_ onto *y* and *E* is the error term that captures all random error, or discrepancies between the outputs predicted by the model and our actual data that are not explained by other factors. In R, the working model is written:

spatial pattern∼species diversity+(1+species diversity|year)+(1+species diversity|cell/zone/quadrat) (6)

Here, spatial pattern equates to *y* (Eq 5), species diversity equals *X*, the term (1 + species diversity | *year*) equals *Z*_1_, and (1 + species diversity | *cell/zone/quadrat*) equals *Z*_2_. Random effects are written (1 + species diversity | *x*) in the working model, rather than (1 | *x*) as they are expressed in the null model, in order to allow for interactions between fixed species diversity effects and random effects.

It is important to note that spatial metrics are often highly correlated with one another [34, 35], which can interfere with interpreting relationships between spatial metrics and other variables. Therefore, before examining relationships between plant species diversity and spatial metrics, we looked for significant relationships among spatial metrics. We deemed SHDI to be a fundamental variable of interest, as we felt it was most likely to correlate with diversity metrics due to its conceptual similarity to SWD. Rather than assess relationships between all possible combinations of spatial variables, we therefore only regressed SHAPE_AM and CONTAG on SHDI in order to gauge relationships among the spatial metrics we had selected. We used altered versions of the working and null models, in which SHDI, rather than species diversity, was the independent variable.

Specifically, to test for potential correlations of the spatial variables, we ran the working and null models for each combination of the other spatial metrics and SHDI (SHAPE_AM v. SHDI and CONTAG v. SHDI) and conducted a Likelihood Ratio Test [36] between the two models for each combination using ANOVA to determine the statistical significance of fixed SHDI effects on SHAPE_AM and CONTAG. We found that SHDI had no significant correlation with either SHAPE_AM (p > 0.5) or CONTAG (p > 0.5). Therefore, we conclude that these three metrics characterize sufficiently distinct aspects of spatial pattern that they can be considered independently.

We then ran the working and null models for all combinations of spatial and diversity metrics (i.e., native SWD v. SHDI, FQAI v. CONTAG, etc.) and conducted a Likelihood Ratio Test [36] to determine the statistical significance of fixed plant spatial effects on species diversity. Because different variables were measured in different units, comparison of numeric values of independent variable coefficients (*X*) does not provide a meaningful measure of the relative strengths of different relationships (Eq 5). For this reason, our results include only the directions and significance of relationships (positive or negative slope), rather than the magnitude of coefficients. We do report the standard error associated with each estimate of independent variable coefficient, as well as the t-value for each estimate. We also report the AICc (Akaike Information Criterion) value for each relationship, which is a statistic that assesses how well the model explains the relationship under examination based on the trade-off between the model’s goodness-of-fit and its complexity. In our initial development of the working model, we compared AICc values of several models to find the one that best explained our data.

## Results

We found that on-the-ground measures of plant species diversity significantly affected some spatial metrics but not others. Native SWD had a significant positive effect on SHDI (p < 0.01; Table 3). Likewise, FQAI had a significant positive effect on SHDI (p < 0.01; Table 3). FQAI also had a significant positive effect on CONTAG (p < 0.01), but native SWD had a significant slightly negative effect on CONTAG (p < 0.01; Table 3). SHAPE_AM was not significantly affected by either native SWD (p = 0.09) or FQAI (p = 0.23). Bivariate scatterplots of significant relationships are included as S1 Fig.

**Table 3:** Significant relationships between species diversity and spatial metrics.

Relationship	Direction of Relationship and Significance	Standard Error of Estimate	T-value	AICc
Native SWD v. SHDI	+ (p < 0.01)	0.06	7.14	140.11
FQAI v. SHDI	+ (p < 0.01)	0.01	4.36	189.34
Native SWD v. CONTAG	- (p < 0.01)	4.97	-0.01	2417.42
FQAI v. CONTAG	+ (p < 0.01)	0.66	1.95	2423.35

## Discussion

We investigated relationships between plant spatial pattern and plant species diversity over a five year period in experimental wetland systems that had been intentionally managed to produce different levels of plant species diversity. We observed that greater values of on-the-ground plant species diversity (both native SWD and FQAI) positively affected SHDI. This relationship makes intuitive sense, since SHDI is basically a variation of SWD intended to capture the diversity of cover types; most cover types in our analysis represent individual plant species. While perhaps unsurprising, this finding is nevertheless of potential importance. The fact that strong relationships exist between remotely sensed data with much fewer plant cover type categories (11 cover types) and on-the-ground measures of plant species diversity (98 species) suggests the potential utility of high-resolution remote sensing as a robust tool for estimating plant species diversity in wetlands. Broad scale remote sensing has previously been used to assess certain aspects of wetland restoration success (e.g., [37]). With the abundance of high-resolution satellite imagery available for many places, and with rapid technological advances in the resolution of remotely sensed data now available using drones, analysis of imagery could prove to be a more efficient method than labor- and time-intensive on-the-ground diversity surveys.

It is a bit harder to immediately reconcile our finding that that CONTAG was positively affected by FQAI but negatively affected by native SWD. This difference in the relationships observed between CONTAG and our two measures of species diversity could lie in the nuances of what these two metrics actually measure. A primary difference between the two diversity metrics is that SWD considers species evenness (i.e., how evenly abundant different species are), while FQAI does not. Furthermore, FQAI is weighted to value certain species over others based on their rarity, while SWD is not. Other studies have also found both positive and negative relationships between plant species diversity and landscape-level metrics of spatial heterogeneity (e.g., [38, 39]). The fact that the two measures of plant species diversity examined in this study exhibit distinct relationships with spatial metrics examined drives home the need for additional theoretical and empirical work to better define such relationships. Ultimately, additional studies that assess and compare on-the-ground measures of plant diversity with remotely sensed spatial data, conducted in multiple different types of ecosystems and at multiple sales, have the potential to deepen our understanding of relationships between diversity, spatial patterns and ecological function.

## Supporting Information

S1 Fig Bivariate scatterplots of significant relationships between species diversity and spatial metrics. (TIF)
