# Supplementary figures and images for: Relationships between Spatial Metrics and Plant Diversity in Constructed Freshwater Wetlands
Source: PLoS One. 2015 Aug 21;10(8):e0135917. doi: 10.1371/journal.pone.0135917 (PMC4546644; doi:10.1371/journal.pone.0135917)

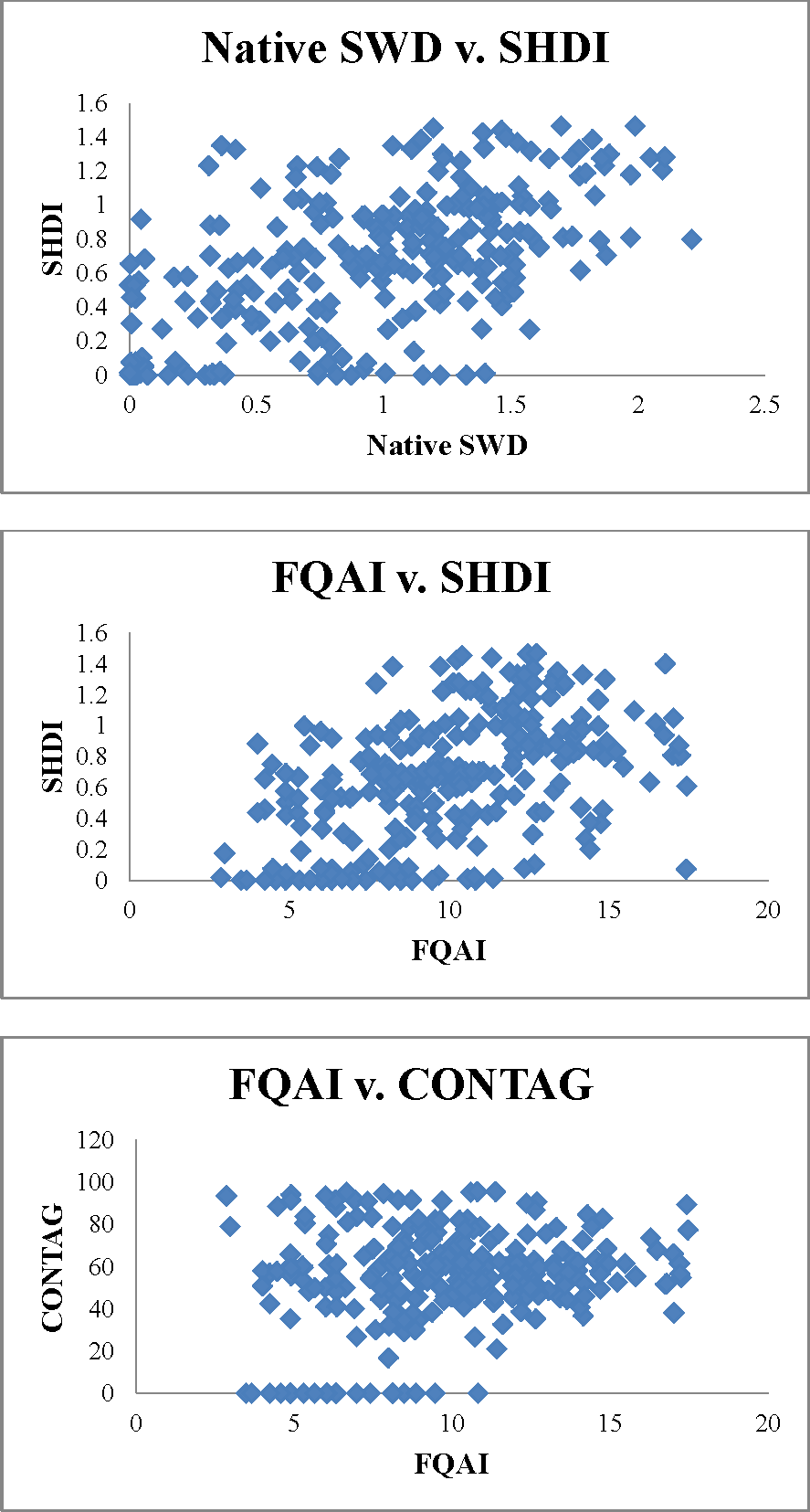

Supplement: S1 Fig — (TIF) [file pone.0135917.s001.tif]
